# Births and Deaths in Philadelphia, in 1859

**Published:** 1860-09

**Authors:** 


BIRTHS AND DEATHS IN PHILADELPHIA, IN 1859.—The total number of births in this city during the last year was 14,832, of which 7669 were males, and 7163 were females.

The number of deaths within the same period was 9742, of which 5160 were males, and 4582 were females. Of the above, deducting the still-born, of whom there were 658, there were males, of 20 years and upwards, 1917; under 20 years, 2508; of females, of 20 years and upwards, 1699; under 20 years, 2249. The above list comprises 1505 deaths by pulmonary phthisis; 544 by inflammation of the lungs; 520 by convulsions; 408 by cholera infantum; 366 by marasmus; 267 by typhoid fever; 234 by dropsy of the brain; and 330 by inflammation of the brain. The mortality from cancer was 132.

